# The backbone symptoms of depression: a network analysis after the initial wave of the COVID-19 pandemic in Macao

**DOI:** 10.7717/peerj.13840

**Published:** 2022-09-15

**Authors:** Yan-Jie Zhao, Wei Bai, Hong Cai, Sha Sha, Qinge Zhang, Si Man Lei, Ka-In Lok, Ines Hang Iao Chow, Teris Cheung, Zhaohui Su, Lloyd Balbuena, Yu-Tao Xiang

**Affiliations:** 1 Unit of Psychiatry, Department of Public Health and Medicinal Administration, & Institute of Translational Medicine, Faculty of Health Sciences, University of Macau, Macao SAR, China; 2 Centre for Cognitive and Brain Sciences, University of Macau, Macao SAR, China; 3 Institute of Advanced Studies in Humanities and Social Sciences, University of Macau, Macao SAR, China; 4 The National Clinical Research Center for Mental Disorders & Beijing Key Laboratory of Mental Disorders, Beijing An Ding Hospital, Beijing, China; 5 Faculty of Education, University of Macau, Macau SAR, China; 6 Kiang Wu Nursing College of Macau, Macau SAR, China; 7 School of Nursing, Hong Kong Polytechnic University, Hong Kong SAR, China; 8 Center on Smart and Connected Health Technologies, Mays Cancer Center, School of Nursing, UT Health San Antonio, San Antonio, Texas, US; 9 Department of Psychiatry, University of Saskatchewan, Saskatoon, Saskatchewan, Canada

**Keywords:** Depression, Survey, Network analysis, COVID-19, Macao

## Abstract

**Background:**

The coronavirus disease 2019 (COVID-19) pandemic disrupted the working lives of Macau residents, possibly leading to mental health issues such as depression. The pandemic served as the context for this investigation of the network structure of depressive symptoms in a community sample. This study aimed to identify the backbone symptoms of depression and to propose an intervention target.

**Methods:**

This study recruited a convenience sample of 975 Macao residents between 20th August and 9th November 2020. In an electronic survey, depressive symptoms were assessed with the Patient Health Questionnaire-9 (PHQ-9). Symptom relationships and centrality indices were identified using directed and undirected network estimation methods. The undirected network was constructed using the extended Bayesian information criterion (EBIC) model, and the directed network was constructed using the Triangulated Maximally Filtered Graph (TMFG) method. The stability of the centrality indices was evaluated by a case-dropping bootstrap procedure. Wilcoxon signed rank tests of the centrality indices were used to assess whether the network structure was invariant between age and gender groups.

**Results:**

Loss of energy, psychomotor problems, and guilt feelings were the symptoms with the highest centrality indices, indicating that these three symptoms were backbone symptoms of depression. The directed graph showed that loss of energy had the highest number of outward projections to other symptoms. The network structure remained stable after randomly dropping 50% of the study sample, and the network structure was invariant by age and gender groups.

**Conclusion:**

Loss of energy, psychomotor problems and guilt feelings constituted the three backbone symptoms during the pandemic. Based on centrality and relative influence, loss of energy could be targeted by increasing opportunities for physical activity.

## Introduction

At the end of 2019, China first reported novel coronavirus pneumonia cases in Wuhan (World Health Organization, 2020b) and it was named the coronavirus disease 2019 (COVID-19) in February 2020 (World Health Organization, 2020a). In order to contain the COVID-19 outbreak and control health-related hazards rapidly and effectively, governments at national and regional levels implemented some travel bans and quarantine regulations (Centers for Disease Control & Prevention, 2021; GOV.UK, 2021; TRAVELBANS, 2021). For cities reliant on tourism, travel restrictions and quarantine regulations would inevitably lead to large-scale economic difficulties and labor market rigidity (Mulder, 2020; Sbai, 2020; Uğur & Akbıyık, 2020; World Tourism Organization, 2020). As the casino capital of the world, Macao may have suffered more than other cities (Direcção dos Serviços de Estatística e Censos, 2021a, 2021b). Economic disruptions are known to trigger mental health problems such as depression (Ennis, Hobfoll & Schröder, 2000; Madianos et al., 2011), anxiety (Crayne, 2020), or post-traumatic stress symptoms (Ennis, Hobfoll & Schröder, 2000).

An online survey of Macao residents reported that 7.7% of the participants suffered from significant financial losses and 11.5% of them had severe depressive symptoms (Macao Daily News, 2021b). It has been confirmed that even without meeting the diagnostic criteria for major depressive disorder (MDD), having depressive symptoms is associated with adverse consequences, such as increased dysfunction (Geiselmann & Bauer, 2000), lower quality of life (Bertha & Balázs, 2013), and an increased risk for suicidal behaviors (Balázs et al., 2013; Hegerl, 2016). For example, a street survey in Macao reported that about 26% of young participants had suicidal ideation in the past 3 months (Macao Daily News, 2021a). The COVID-19 pandemic served as the context for this study of depressive symptoms (“depression” hereinafter) among residents in Macao, with the aim of identifying a possible intervention target.

Traditionally, research about the epidemiology and etiology of major depression follows the latent factor approach (Everett, 2013). This approach postulates that depression is the unobserved entity that is manifested by observable symptoms called indicators (Schmittmann et al., 2013). In this view, depression is the common cause of the observed symptoms, and without it, the symptoms would have zero correlations with each other (Brown, 2006; Everett, 2013; Schmittmann et al., 2013). This is contradicted by the finding that anhedonia, hopelessness or tiredness usually correlated with each other even when the diagnostic criteria for MDD were not fulfilled (Borsboom, 2008; Santos et al., 2017). This implies that the traditional approach may inadequately describe the complexity and dynamics of depression (Marchetti, 2018; Mullarkey, Marchetti & Beevers, 2019).

An alternative approach, called network analysis, focuses instead on symptoms and their relations, without assuming that a latent factor causes the symptoms (Borsboom & Cramer, 2013). Unlike the latent variable approach, the symptoms are considered “active ingredients of mental disorders” instead of mere by-products (Borsboom & Cramer, 2013). Although the debate regarding the pros and cons of each approach continues (Epskamp, Rhemtulla & Borsboom, 2017; Guyon, Falissard & Kop, 2017), network analysis provides insight into how these symptoms are associated and interconnected, and what symptoms can serve as prevention or intervention targets (Borsboom & Cramer, 2013; Cramer et al., 2016; Cramer et al., 2010; Epskamp, Rhemtulla & Borsboom, 2017).

Although several studies using the network approach have probed the association patterns of various psychiatric conditions among different subpopulations during the COVID-19 pandemic (Bai et al., 2021; Di Blasi et al., 2021; Li et al., 2022; Wang et al., 2020), few have focused on tourism-dependent areas like Macao. Previous studies revealed that the network structure of mental health symptoms in various subpopulations might differ by age, gender, or health status (Hartung et al., 2019; Shim et al., 2020; Wang et al., 2020). For this reason we examined age and gender as factors for subgroup analyses.

Examining the interaction of depression symptoms was undertaken with three specific aims: (1) to describe directional relationships among depressive symptoms; (2) to compare the pattern of symptoms across gender and age groups; and (3) to identify potential prevention or intervention targets.

## Methods

### Setting and participants

This cross-sectional study was conducted from August 20 to November 9, 2020, after the initial wave of the COVID-19 pandemic in Macao. The initial wave is defined as the interval between the first recorded COVID-19 case (January 22, 2020) and the last recorded local case of the first wave (March 28, 2020). (Macau Daily, 2020; Tencent, 2021). From March 29, 2020 to August 2, 2021, there were no local infection but only imported cases (BBC News, 2021; Macau Daily, 2021). Participants were invited to participate in an online survey through advertisements in major social network platforms (specifically: WeChat, Facebook, and Instagram). To be eligible, participants had to be: (1) residing in Macao during the COVID-19 pandemic; (2) 18 years of age or above; (3) able to read and understand Chinese; and (4) provided informed consent. The study protocol was approved by the Institutional Review Board (IRB) of Beijing Anding Hospital, Capital Medical University.

### Measures

Depressive symptoms were assessed with the self-rated Patient Health Questionnaire - 9 items (PHQ-9), Chinese version (Wang et al., 2014; Xu, Wu & Xu, 2007). The PHQ-9 asks about nine symptoms: anhedonia, sadness, sleep, energy, appetite, guilt, concentration, psychomotor changes, and suicidal ideation (Kroenke, Spitzer & Williams, 2001; Spitzer, Kroenke & Williams, 1999). These are rated from 0 (not at all) to 3 (nearly every day), with higher scores indicating more severe symptoms. The Chinese version of PHQ-9 is psychometrically validated and has good properties (Xu, Wu & Xu, 2007; Yu et al., 2012). PHQ-9 was the chosen instrument because its items directly correspond to the symptoms of the Diagnostic and Statistical Manual of Mental Disorders (DSM).

### Network analysis

**Figure 1:**
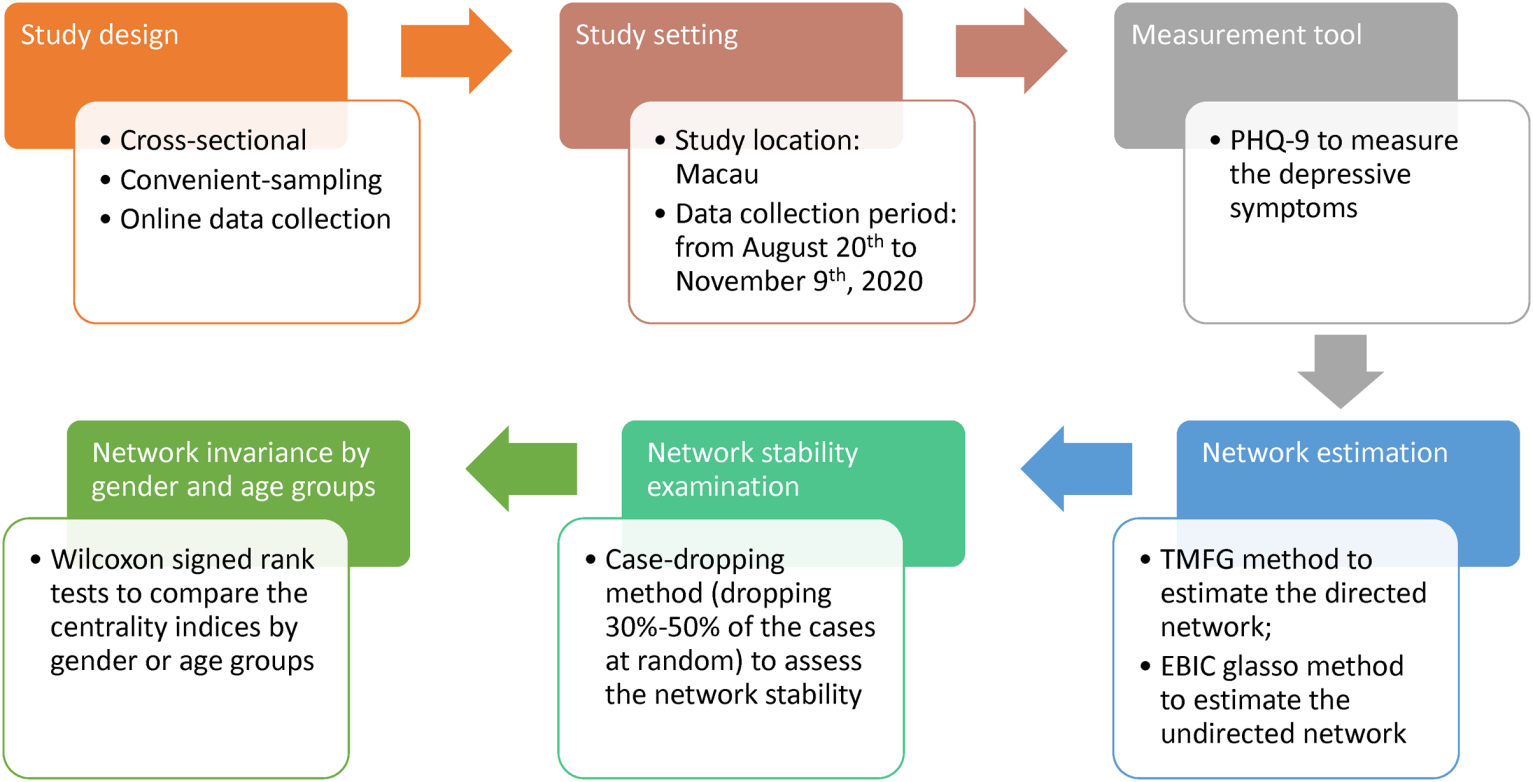
Study procedure flow.

First, the data were verified if they are suitable for network analysis by checking item distributions. All items were not normally distributed so partial polychoric correlations were calculated (Beard et al., 2016). These partial correlations were used to estimate a directed, and weighted network using the Triangulated Maximally Filtered Graph (TMFG) method (Massara, Di Matteo & Aste, 2017). TMFG method distinguishes influencing and influenced nodes based on the concept of node dependence (Jacob et al., 2016). To identify important symptoms, three centrality measures were examined: strength, betweenness, and closeness. For each symptom, the outgoing and incoming strength were compared (Kenett et al., 2010; Kenett et al., 2011). The difference between outgoing and incoming strength is known as node influence (or relative importance), with higher values indicating greater influence (Christensen, 2018). Subsequently, the stability of the centrality indices was checked by dropping between 30–50% of cases. Finally, the indices were checked if they were invariant by age group (≤28 years *vs*. ≥29 years) and gender (Epskamp, Borsboom & Fried, 2018). Please refer to Table 1 for a description of the network properties of interest, what they represent, and how they are calculated. The pattern of symptom relations in the directed network was further validated by comparing it to an undirected network estimated by the extended Bayesian information criterion (EBIC) graphical lasso (glasso) method. Figure 1 shows the procedure flow of this study.

**Table 1:** Directed Network properties and their calculation.

Network property	Intuitive meaning	Calculation
1. Node centrality	More important nodes are central, less important nodes are peripheral	
a. In-(out-) strength	How well a node is directly connected to other nodes (Epskamp, Borsboom & Fried, 2018)	*In-strength:* the sum of the weighted number of arcs (an edge with direction) *ending* in a given node (Guo et al., 2011). *Out-strength:* the sum of the weighted number of arcs *starting* from a given node (Guo et al., 2011).
b. Betweenness	How often a node lies in the shortest path between pairs of nodes (Freeman, 1978) or being “in the middle” of other nodes (Hansen, Shneiderman & Smith, 2011).	*Betweenness of node X* is: 1/(n−1)(n−2) × sum of number of shortest arcs/number of arcs that pass through node X (Rubinov & Sporns, 2010).
c. Closeness	How easy it is for a given node to reach other nodes without relying on intermediary nodes (Leavitt, 1951)	*Closeness of node X* is: The reciprocal of the average distance (with direction) between X and all other nodes (Rubinov & Sporns, 2010).
2. Relative influence	The relative degree of incoming and outgoing edges	Positive values indicate higher outgoing strength relative to incoming strength. Negative values indicate higher incoming strength relative to outgoing strength (Christensen, 2018).
3. Stability of centrality indices	How well the network structure is preserved when taking subsets of cases.	Subsamples of 70%, 60% and 50% were randomly drawn from the full sample, each with 1,000 replications. The ranking of centrality indices was averaged over replications and correlated to their ranking in the full sample (Epskamp, Borsboom & Fried, 2018).
4. Invariance of centrality indices by age (28 years and below *vs*. 29 and above) and gender groups	Whether the important symptoms differ by age or gender	Separate networks were developed by age group and gender. Then, the hypothesis that the ranks of the PHQ-9 symptoms were similar between groups was tested using the paired Wilcoxon sign rank test.

All analytical procedures were performed in the R language (R Core Team, 2020) using the packages bootnet, networktools, NetworkToolbox, psych, and qgraph (Christensen, 2018; Epskamp, Borsboom & Fried, 2018; Epskamp et al., 2012; Jones, 2020; Revelle, 2020).

## Results

In all, 975 persons participated (women: 65.0%, 31.1 ± 11.5 years; men: 35.0%, 27.8 ± 10.0 years) in the survey. Most participants were well-educated (*i.e*., undergraduate/college or higher: 78.5%) and were unmarried (61.4%).

### Node centrality and node influence

**Figure 2:**
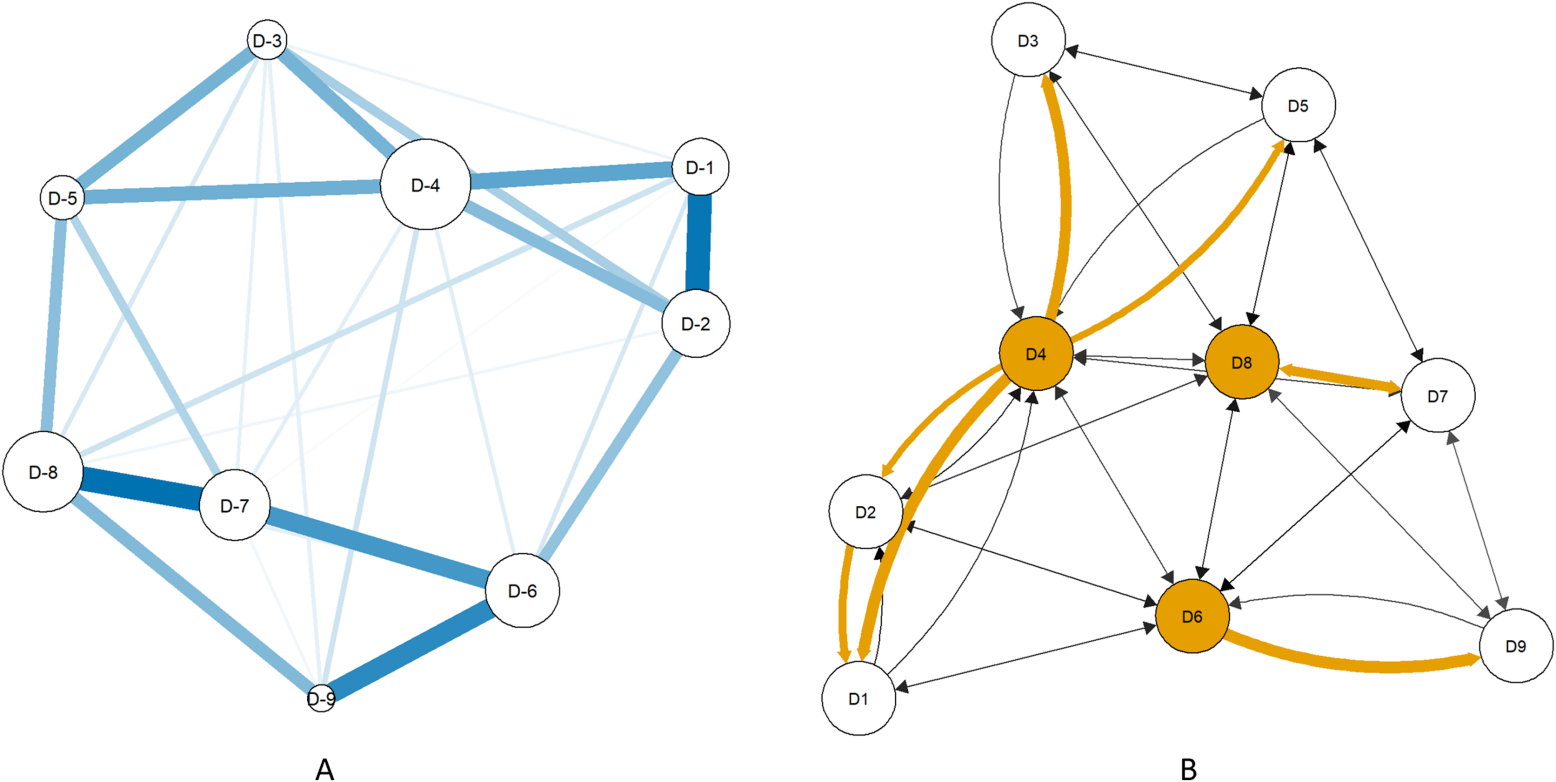
PHQ-9 symptom networks estimated by the extended Bayesian information criterion graphical lasso method (A) and the Triangulated Maximally Filtered Graph method (B). Key: D1: Anhedonia; D2: Sad mood; D3: Sleep problems; D4: Loss of energy; D5: Appetite change; D6: Guilt feelings; D7: Difficulty in concentration; D8: Psychomotor problems; D9: Suicidal ideation. Figure 2A: Node size indicates strength centrality and edge thickness indicates weight. Figure 2B: Shaded nodes have the highest outgoing strength. Arrows indicate the direction of influence. Yellow arrows indicate strong associations.

Loss of energy, psychomotor problems, and guilt feelings were identified as the central items in strength (outgoing and incoming), betweenness, and closeness. The same symptoms had the most influence except that concentration difficulty was ranked higher than guilt feelings (Table 2). Loss of energy, psychomotor problems, guilt feelings, difficulty in concentration had positive relative influence values (Table 2). Figure 2A displays the undirected network of PHQ-9 symptoms, and Figure 2B displays the directed network of PHQ-9 symptoms. The directed graph showed that loss of energy had the highest number of outward projections to other symptoms (Figure 2B).

**Table 2:** Centrality measures of the nine depressive symptoms (sorted in descending order of outgoing strength).

PHQ-9 symptom	Strength			Betweenness	Closeness
	Outgoing	Incoming	Relative influence		
D4: Loss of energy	3.05	2.61	0.08	12	4.09
D8: Psychomotor problems	2.82	2.60	0.04	6	4.14
D6: Guilt feelings	2.43	2.35	0.02	8	3.88
D7: Difficulty in concentration	2.09	1.95	0.04	2	3.55
D2: Sad mood	1.61	1.66	−0.02	2	3.35
D5: Appetite change	1.48	1.67	−0.06	0	3.43
D1: Anhedonia	1.22	1.317	−0.04	0	3.13
D3: Sleep problems	1.06	1.25	−0.08	0	3.16
D9: Suicidal ideation	0.98	1.324	−0.15	0	3.20

### Stability of centrality indices

The correlation of the symptom ranks in each centrality index between the subsamples and the full sample was 0.71 or higher (Table 3), indicating that the network structure remained stable after randomly dropping up to 50% of the study sample. (Epskamp, Borsboom & Fried, 2018)

**Table 3:** Rank correlations of centrality indices by the case-dropping method.

	70% subsample retained	60% subsample retained	50% subsample retained
In-strength	0.75	0.76	0.76
Out-strength	0.93	0.93	0.93
Betweenness	0.95	0.94	0.95
Closeness	0.71	0.71	0.72

### Invariance of centrality indices

**Figure 3:**
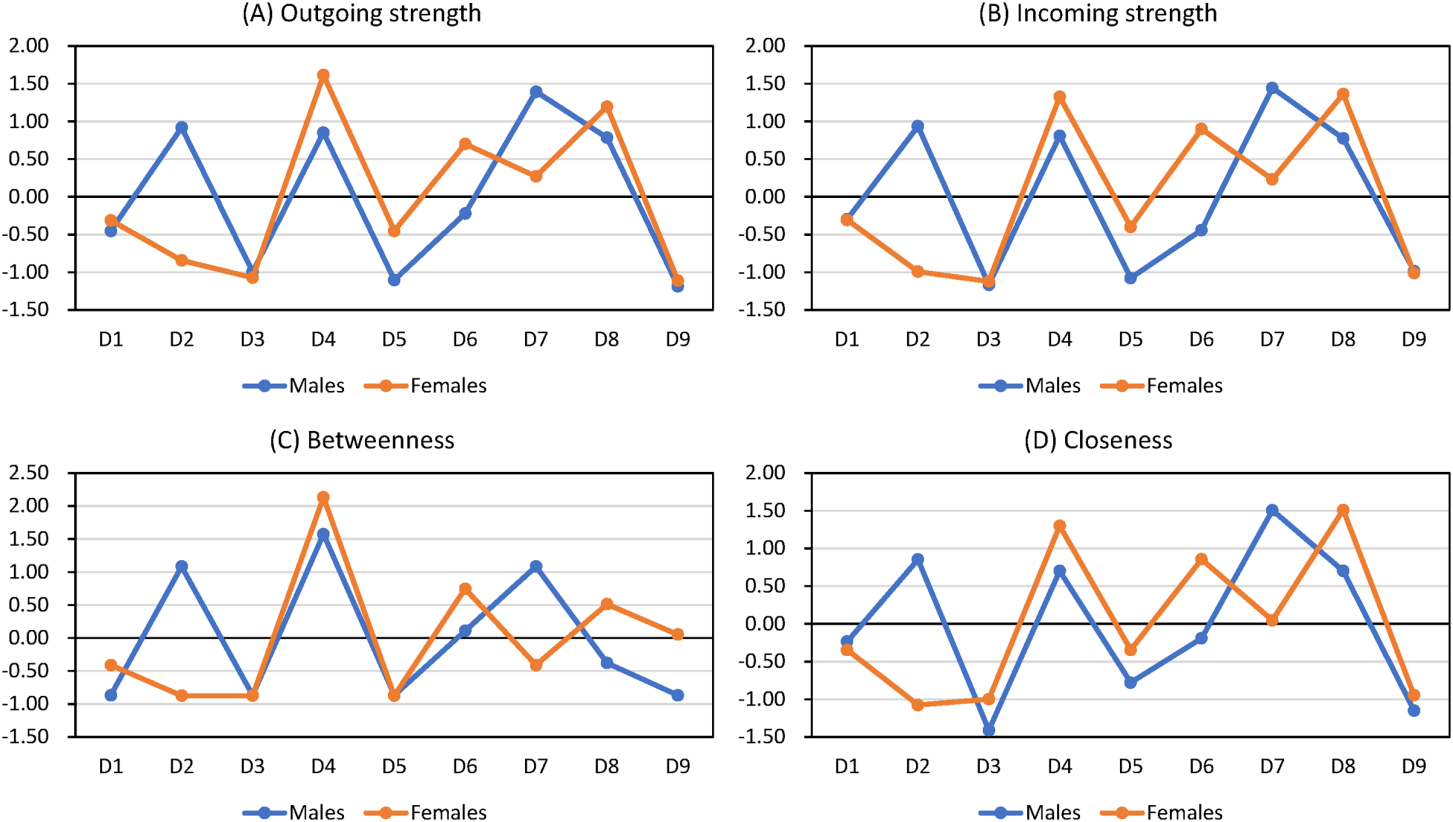
Centrality indices of PHQ-9 symptoms by gender (z-score transformed).

**Figure 4:**
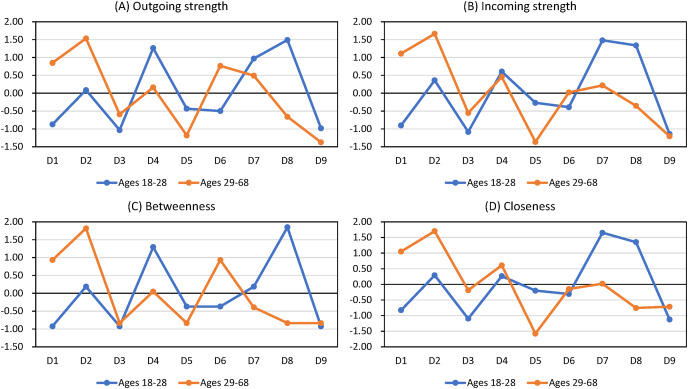
Centrality indices of PHQ-9 symptoms by age group (z-score transformed).

The Wilcoxon signed rank tests showed that outgoing strength, incoming strength, betweenness, and closeness did not vary by age or gender groups (all *p* values > 0.05; Tables 4 and 5). Figure 2 shows that the central symptoms are the same in undirected and directed networks (Figure 2A and 2B). Figures 3 and 4 show the z-score-transformed centrality indices of PHQ-9 symptoms by gender and by age group.

**Table 4:** Centrality indices of PHQ-9 symptoms by gender.

	Outgoing strength		Incoming strength		Betweenness		Closeness	
	Males	Females	Males	Females	Males	Females	Males	Females
D1: Anhedonia	1.57	1.60	1.74	1.68	0	2	3.47	3.38
D2: Sad mood	2.58	1.18	2.42	1.31	8	0	3.97	3.10
D3: Sleep problems	1.17	1.00	1.26	1.24	0	0	2.93	3.13
D4: Loss of energy	2.53	3.12	2.35	2.56	10	13	3.90	4.01
D5: Appetite change	1.09	1.49	1.31	1.63	0	0	3.22	3.38
D6: Guilt feelings	1.74	2.40	1.66	2.33	4	7	3.49	3.84
D7: Difficulty in concentration	2.93	2.06	2.70	1.97	8	2	4.27	3.53
D8: Psychomotor problems	2.48	2.79	2.33	2.58	2	6	3.90	4.09
D9: Suicidal ideation	1.03	0.97	1.36	1.30	0	4	3.05	3.15
Wilcoxon’s signed rank test	W^+^ = 22 W^−^ = 23 *p* = 1.0		W^+^ = 23 W^−^ = 22 *p* = 1.0		W^+^ = 13 W^−^ = 15 *p* = 1.0		W^+^ = 18 W^−^ = 27 *p* = 0.64	

**Note:**

W^+^: the sum of positive differences between male and female scores, W^−^: the sum of negative differences between male and female scores.

**Table 5:** Centrality indices of PHQ-9 symptoms by age group.

	Outgoing strength		Incoming strength		Betweenness		Closeness	
	Ages 18–28	Ages 29–68	Ages 18–28	Ages 29–68	Ages 18–28	Ages 29–68	Ages 18–28	Ages 29–68
D1: Anhedonia	1.10	2.62	1.31	2.56	0	8	3.03	4.15
D2: Sad mood	1.82	3.12	1.93	2.84	4	12	3.44	4.46
D3: Sleep problems	0.98	1.57	1.22	1.72	0	0	2.93	3.56
D4: Loss of energy	2.70	2.12	2.05	2.23	8	4	3.43	3.94
D5: Appetite change	1.43	1.14	1.62	1.31	2	0	3.26	2.90
D6: Guilt feelings	1.38	2.56	1.56	2.01	2	8	3.22	3.58
D7: Difficulty in concentration	2.48	2.36	2.48	2.11	4	2	3.94	3.66
D8: Psychomotor problems	2.87	1.52	2.41	1.82	10	0	3.83	3.29
D9: Suicidal ideation	1.02	1.00	1.19	1.39	0	0	2.92	3.31
Wilcoxon’s signed rank test	W^+^ = 18 W^−^ = 27 *p* = 0.64		W^+^ = 14 W^−^ = 31 *p* = 0.34		W^+^ = 13 W^−^ = 15 *p* = 0.93		W^+^ = 9.5 W^−^ = 35.5 *p* = 0.12	

**Note:**

W^+^: the sum of positive differences between male and female scores, W^−^: the sum of negative differences between male and female scores.

## Discussion

This study found that loss of energy, psychomotor problems, and guilt feelings are the most important symptoms among Macao residents after the initial wave of COVID-19. The results remained the same even when the following factors were varied: (i) sample size, (ii) age or gender, or (iii) estimation method (directed or undirected).

The finding that loss of energy has the highest strength centrality, indicates that it is the nexus of depressive symptoms in community-based setting, which is consistent with another network analysis conducted in mainland China (Wang et al., 2020). Loss of energy refers to a feeling of tiredness, fatigue or having little energy (Billones, Kumar & Saligan, 2020), and it is a very prevalent symptom in depression (Angst & Dobler-Mikola, 1984; Buchwald & Rudick-Davis, 1993; Corfield, Martin & Nyholt, 2016; Pae et al., 2007; Reyes-Gibby et al., 2006), especially in people who somatize their depressive symptoms, a tendency found in East Asians (Stahl, 2002). The Hinz et al. (2016) study revealed that loss of energy had the highest factor loading in the PHQ-9, confirming that loss of energy is a core symptom of depression. Previous neuronal pathway studies also showed that chronic fatigue and depression shared the same neurobiological mechanisms (Berman et al., 1999; Chaves-Filho et al., 2019).

In a community-based setting, previous studies reported that loss of energy is an early-endorsed symptom when people encounter some depressing or stressful life events (Fava & Tossani, 2007; Pae et al., 2007; Robinson et al., 2015), and loss of energy is predictive for an increased risk of future depression (Demyttenaere, De Fruyt & Stahl, 2005), which could explain the high centrality of energy loss in this study. However, in psychiatric samples, the top-ranked central symptom is usually sad mood (Beard et al., 2016; van Rooijen et al., 2018), which is different from the finding in this study. This suggests a discrepancy between psychiatric and community samples. Also, Borsboom’s group ran network analysis with the nine DSM depression symptoms combined with 19 other non-DSM symptoms of depression in a group of depressed outpatients (Fried et al., 2016). Interestingly, loss of energy was the most central symptom by strength in their expanded list of symptoms, suggesting that loss of energy could be a symptom that transcends cultural boundaries. Since the present study was conducted during the pandemic, energy loss is probably related to the physical inactivity induced by the lockdown experience (McIlvenny et al., 2000; Stewart et al., 1998), and the worries brought about by unemployment and reduced income (Sharpe & Wilks, 2002).

What the present study adds to the literature is that targeting loss of energy may prevent full-blown major depression or alleviate it if already diagnosed. In particular, the results show that appetite change, sleep problems, anhedonia, and sadness are more strongly influenced by loss of energy than any other symptoms. This implies that treating loss of energy, whether by lifestyle changes or medications, may sever the backbone that projects to four other symptoms. Given that loss of energy is very prevalent in major depression (Angst & Dobler-Mikola, 1984; Buchwald & Rudick-Davis, 1993; Corfield, Martin & Nyholt, 2016; Pae et al., 2007; Reyes-Gibby et al., 2006), targeting loss of energy may have substantial payoff.

Engaging in physical activity during the pandemic was inversely related to major depression symptoms in older adults (Callow et al., 2020). Similarly, adolescents in China who engaged in more than 30 mins of physical activity daily were less prone to depressive symptoms (Kang et al., 2021). This highlights one of the numerous trade-offs between infection control and mental health during the COVID-19 pandemic.

Psychomotor disturbance was the second most central symptom in this study and this is consistent with previous studies (Ge et al., 2019; Wang et al., 2020). Psychomotor abnormalities, such as slowing down in thought, motor retardation, restlessness or fidgeting, are manifestations of concentration difficulty, anxiety or mental tension (Bennabi et al., 2013). Slowness in thoughts and behaviors is one of the primary manifestations in depression according to the DSM 5^th^ edition (American Psychiatric Association, 2013). Fried et al. (2015) showed that when people deal with stressful events, they tend to endorse psychomotor disturbance the highest, although this symptom is not very frequently endorsed in depression (Lamers et al., 2012).

It is also notable that loss of energy and psychomotor disturbance were the two most central symptoms. Both the International Classification of Diseases (ICD) and the DSM systems consider sadness and anhedonia as the two core symptoms (Kennedy, 2008), implying that depression is primarily a cognitive and affective disorder (Demyttenaere, De Fruyt & Stahl, 2005). This suggests that if sadness and anhedonia were treated, psychomotor disturbances and loss of energy will resolve. However, loss of energy tends to linger as a residual symptom after the emotional symptoms have resolved (Demyttenaere, De Fruyt & Stahl, 2005). It was also shown that psychomotor retardation responds to high frequency repetitive transcranial magnetic stimulation (rTMS) independently of overall clinical response (Baeken et al., 2010). Psychomotor retardation is associated with high levels of interleukin 6 (IL-6), and decreasing IL-6 levels improved psychomotor retardation (Belge et al., 2021; Goldsmith et al., 2016). In this broader context, the results in this study suggest a treatment (or prevention) regimen that targets energy and motor symptoms.

Guilty feelings had the third highest centrality and is the top-ranked affective symptom. Guilt was described by Klass (1987) as a state that includes a cognitive component of having done something wrong and an affective component of shame even though neither wrongful actions nor other people are passing judgment. Previous studies found that guilt is one of the most useful discriminating symptoms of depression (Ghatavi et al., 2002; Jarrett & Weissenburger, 1990). Excessive guilt could lead to problematic outcomes such as impaired motivation and self-care, possibly evolving to depression (Luck & Luck-Sikorski, 2020; Tilghman-Osborne, Cole & Felton, 2010, 2012). The directed network analysis implies that “people do not feel guilty because they are thinking of suicide (*i.e*., a big sin)” but rather “people are thinking of suicide because they feel they have fallen short of some standard (*i.e*. guilt).” This fits logically with Joiner’s (2005) theory of perceived burdensomeness to loved ones as a motive for suicide. In his theory, suicidal people think of themselves as a liability, so they are better off dead.

Using factor analysis, Berrios et al. (1992) found that guilt consists of cognitive and affective factors. Importantly, they reported that the cognitive factor—the negative self-appraisal was correlated with psychomotor retardation. Although the results in this study showed that guilt and psychomotor problems were not very strongly correlated, their earlier finding and the present study could be the basis for speculating that by targeting motor symptoms, feelings of guilt might also alleviate. There has been much work suggesting that dopaminergic treatments for psychomotor retardation, although the hypothesized downstream benefit is for anhedonia (Argyropoulos & Nutt, 2013; Kapur & Mann, 1992). Guilt has received less attention compared to other emotions such sadness, anxiety, and anger, so it would be important to further study its role in major depression.

The advantages of this study consist of a relatively large sample size and a novel approach (network analysis) in a community whose main economic activity was shut down by the pandemic (Direcção dos Serviços de Estatística e Censos, 2021a, 2021b). However, this study is subject to several limitations. First, as a cross-sectional study it lacks a time dimension for validating the predicted direction among symptoms. Hence, the symptom relations of the network cannot be interpreted as causal. In addition, changes in the pattern of depressive symptoms pre- and post-pandemic could not be studied. Therefore, this study remains exploratory and future longitudinal studies are necessary. Second, both network estimation methods in this study rely on symptoms that are rated on an ordinal scale. These ordinal variables are assumed to be thresholds of a normally distributed variable. However, suicidal ideation (D9), may not be normally distributed in the population (Glashouwer et al., 2010). Third, the analysis in this study limited its scope to the nine canonical symptoms of depression, so including depressive symptoms outside of them might produce a different pattern of relationships. Fourth, this study was conducted in a community sample, hence it cannot be generalized to special populations such as adolescents, the elderly, or psychiatric samples.

## Recommendations

The findings would be important for policy makers and mental health service providers to balance pandemic control regulations with opportunities to engage in physical activity. The promotion of mental health by a more active lifestyle should be encouraged. Targeting loss of energy may help relieve the other depressive symptoms.

## Conclusion

Loss of energy, psychomotor problems and guilt feelings constituted the three backbone symptoms during the pandemic. Based on centrality and relative influence, loss of energy could be targeted by increasing opportunities for physical activity.

## Supplemental Information

10.7717/peerj.13840/supp-1 Supplemental Information 1 Supporting document for free use of PHQ-9.

10.7717/peerj.13840/supp-2 Supplemental Information 2 Reading code.

10.7717/peerj.13840/supp-3 Supplemental Information 3 Raw data.

## Additional Information and Declarations

### Data Availability

The following information was supplied regarding data availability: Raw data and code are available in the Supplemental Files.
