# Supplementary material for: The backbone symptoms of depression: a network analysis after the initial wave of the COVID-19 pandemic in Macao
Source: PeerJ. 2022 Sep 15;10:e13840. doi: 10.7717/peerj.13840 (PMC9482773; doi:10.7717/peerj.13840)
Supplement: Supplemental Information 1 [file peerj-10-13840-s001.pdf]

The Patient Health Questionnaire-9 (PHQ-9) is a brief psychological screening instrument designed to measure symptoms of depression in primary care settings. The PHQ-9 is available to healthcare providers completely free of charge. Pfizer Inc., the legal copyright holder, explicitly states that “no permission is required to reproduce, translate, display or distribute the PHQ-9.”

Here are some evidences supporting the free use of PHQ-9:

https://www.bmedreport.com/archives/14638

https://www.pfizer.com/news/press-release/press-release-detail/pfizer_to_offer_free_public_access_to_mental_health_assessment_tools_to_improve_diagnosis_and_patient_care
